# Comparative efficacy and cognitive safety of bifrontal versus bitemporal electroconvulsive therapy in adolescents with major depressive disorder: a retrospective cohort study

**DOI:** 10.3389/fpsyt.2026.1843108

**Published:** 2026-08-26

**Authors:** Xiaolu Chen, Ke Liu, Xiaoshan Shen, Yifan Yang, Hao Yu, Qibing Chen, Xiaoli Wang, Xiao Li

**Affiliations:** 1 Department of Psychiatry, The First Affiliated Hospital of Chongqing Medical University, Chongqing, China; 2 Department of Emergency, The Second Affiliated Hospital of Chongqing Medical University, Chongqing, China; 3 The First Clinical College, Chongqing Medical University, Chongqing, China; 4 Department of Anesthesiology, The First Affiliated Hospital of Chongqing Medical University, Chongqing, China; 5 Department of Psychiatry, Chongqing Nanchuan District Hospital of Traditional Chinese Medicine, Chongqing, China

**Keywords:** adolescent, bifrontal, bitemporal, cognitive safety, depression, electroconvulsive therapy, suicide

## Abstract

**Background:**

Electroconvulsive therapy (ECT) remains a rapid and highly effective treatment for severe, treatment-resistant depression in adolescents, particularly when immediate suicide risk is present. However, its clinical utilization in the pediatric population is still low, largely restricted by pervasive concerns regarding cognitive impairment. While randomized trials in adults suggest that bifrontal (BF) electrode placement offers a superior cognitive safety profile compared to the standard bitemporal (BT) placement, comparative evidence specifically regarding the developing adolescent brain remains scarce.

**Methods:**

We conducted a retrospective cohort study at the Department of Psychiatry in a tertiary general hospital, analyzing 217 adolescents (aged 12–18 years) diagnosed with major depressive disorder (MDD) who underwent a course of ECT between January 2018 and June 2024. Patients were categorized into the BF group (n = 111) or BT group (n = 106) based on the electrode placement utilized. The primary efficacy outcomes were the response rate (≥50% reduction in 24-item Hamilton Depression Rating Scale (HAMD-24) scores) and remission rate (HAMD-24 score ≤8). Secondary outcomes included the reduction in suicidal ideation (Beck Scale for Suicide Ideation (BSSI) scores), the incidence of subjective memory complaints, and post-ictal recovery time. Multivariate logistic regression was performed to identify independent predictors of subjective memory complaints, adjusting for baseline severity and concurrent medications.

**Results:**

Baseline demographic and clinical characteristics, including severity of depression and suicidality, were well-balanced between groups. The BF group achieved a numerically higher response rate (85.6%) compared to the BT group (82.1%), although this difference did not reach statistical significance (p = 0.478; odds ratio [OR] = 1.29, 95% CI 0.64–2.42). Remission rates were also comparable (33.3% vs. 33.0%; p = 0.963). Both groups showed equal efficacy in reducing suicidal ideation (p > 0.05). While efficacy outcomes were similar, the two modalities showed notable differences in two specific safety-related endpoints that are relevant to daily clinical practice: the incidence of subjectively reported memory complaints (28.8% in the BF group compared with 67.9% in the BT group; p < 0.001; OR = 0.20, 95% CI 0.11–0.36) and the objectively measured post-ictal recovery time (23.7 ± 7.7 min for BF compared with 33.4 ± 9.9 min for BT; p < 0.001; Cohen’s d = 1.12). After adjusting for age, baseline HAMD-24 scores, and concurrent use of benzodiazepines and antipsychotics, BF placement remained a significant independent protective factor against subjective memory complaints (adjusted OR = 0.21, 95% CI: 0.11–0.39, p < 0.001).

**Conclusion:**

In adolescents with MDD, bifrontal ECT showed short-term antidepressant and anti-suicidal outcomes comparable to those of bitemporal ECT in this retrospective cohort. BF ECT appeared to be associated with fewer subjective memory complaints (as assessed by spontaneous patient reports and daily physician inquiries) and shorter post-ictal recovery time. These two endpoints, while clinically relevant in routine practice, do not capture the full spectrum of cognitive function; standardized neuropsychological assessment would be required for a comprehensive comparison. Given the retrospective design and non-random treatment allocation, these findings should be interpreted cautiously and confirmed in prospective controlled studies.

## 1 Introduction

Major depressive disorder (MDD) in adolescence represents a burgeoning public health crisis globally, characterized by distinct phenomenology, a chronic fluctuating course, and a profoundly elevated risk of suicide (1, 2). Suicide remains the second leading cause of death among individuals aged 15–29 years, highlighting the urgent need for rapid and robust interventions (3). While the combination of pharmacotherapy, primarily selective serotonin reuptake inhibitors, and psychotherapy constitutes the first-line treatment, a substantial subset of adolescents—approximately 40%—fail to achieve remission, developing treatment-resistant depression (4). For these severe, refractory, and often life-threatening cases, electroconvulsive therapy (ECT) is recognized by the American Academy of Child and Adolescent Psychiatry (AACAP) as a highly effective, life-saving intervention (5). Despite its established efficacy, the utilization of ECT in the pediatric population is still low compared to adults (6). It is largely driven by persistent stigma and profound concerns among clinicians, parents, and patients regarding cognitive side effects. The most feared adverse events are retrograde amnesia and subjective memory impairment. In the context of adolescence—a critical period for academic learning, identity formation, and psychosocial development—the potential for cognitive disruption is particularly concerning (7). The adolescent brain is undergoing dynamic structural and functional maturation, including synaptic pruning and myelination. The hippocampus, a structure critical for memory and emotion regulation, exhibits significant plasticity during this period, potentially rendering it hypersensitive to the electrical insults associated with traditional ECT protocols (8). Strategies to optimize the risk-benefit ratio of ECT have focused on refining stimulation parameters, such as pulse width, and modifying electrode placement. The traditional “gold standard,” bitemporal (BT) placement, involves positioning electrodes over the temporal regions. While highly effective, this placement may cause current to pass through the temporal lobes and hippocampi to induce a generalized seizure (9). In contrast, bifrontal (BF) placement positions electrodes over the prefrontal cortex. Computational modeling of electric fields suggests that BF placement focuses the current density on the frontal poles, which may shunt current away from the temporal lobes and potentially reduce hippocampal exposure (10, 11). Despite its established efficacy and safety in adults, the evidence base for ECT in adolescents remains limited and primarily derived from small retrospective cohorts or case series. Recent population-based and systematic reviews have confirmed that ECT yielded high response rates (70%–82%) for severe, treatment-resistant depression in adolescents, with a favorable safety profile (4, 12). However, direct comparative data on cognitive safety between bifrontal (BF) and bitemporal (BT) electrode placements—the two most widely used techniques—remain scarce in the adolescent population. In adult populations, a robust body of evidence, including randomized controlled trials, has demonstrated that BF ECT results in superior cognitive outcomes compared to BT ECT without compromising antidepressant efficacy (15, 16). However, pediatric ECT research has historically relied on data extrapolated from adult studies. There is a lack of direct comparative studies investigating whether the “cognitive sparing” advantage of BF placement holds true for the developing adolescent brain. This gap is critical because if BF placement is indeed safer, maintaining the continued use of BT placement as a default in adolescents could be ethically questionable (17). We aimed to conduct a large-scale retrospective cohort study to directly compare the clinical efficacy, specifically focusing on antidepressant and anti-suicidal effects, and the safety profile of BF versus BT ECT in adolescents. We hypothesized that BF ECT would demonstrate similar clinical efficacy while significantly reducing the burden of cognitive complaints compared to traditional BT placement.

## 2 Methods

### 2.1 Study design and participants

This was a single-center, retrospective cohort study conducted at the Department of Psychiatry, The First Affiliated Hospital of Chongqing Medical University. The study protocol was approved by the Institutional Review Board of the hospital (no. 2017-157). Written informed consent was obtained from all patients and/or their legal guardians prior to the commencement of ECT treatment, which included consent for the retrospective use of their anonymized clinical data for research purposes. The study was conducted in accordance with the Declaration of Helsinki. We systematically reviewed the electronic medical records of all inpatients admitted between January 2018 and June 2024.

The inclusion criteria were defined as follows: (1) age between 12 and 18 years inclusive at the time of the first treatment—an age range consistent with published adolescent ECT studies and with the AACAP position that clinical indication, not age alone, should determine eligibility for ECT (5, 12); (2) a primary discharge diagnosis of Major Depressive Disorder (MDD), with or without psychotic features, according to the Diagnostic and Statistical Manual of Mental Disorders, Fifth Edition (DSM-5) (18); (3) completion of an acute course of ECT, defined as receiving a minimum of 6 sessions. This threshold aligns with the NICE guidance, which states that a course of ECT typically consists of 6–12 treatments, and with the 2017 Chinese ECT expert consensus recommending 6–12 sessions per course (19). Six sessions are widely accepted as the minimum number to assess initial treatment response in clinical practice and published ECT studies. The mean of approximately 10 sessions in this cohort was determined by clinical response; ECT was discontinued when the treating psychiatrist, in consultation with the patient and guardians, determined that sufficient improvement had been achieved in depressive symptoms and suicidality, or when the patient or guardian declined further treatment. We did not restrict inclusion to a strictly defined treatment-resistant depression (TRD) sample, because the operational definition of TRD in adolescents varies across studies and no universal consensus has yet emerged (20, 21).

**Figure 1:**
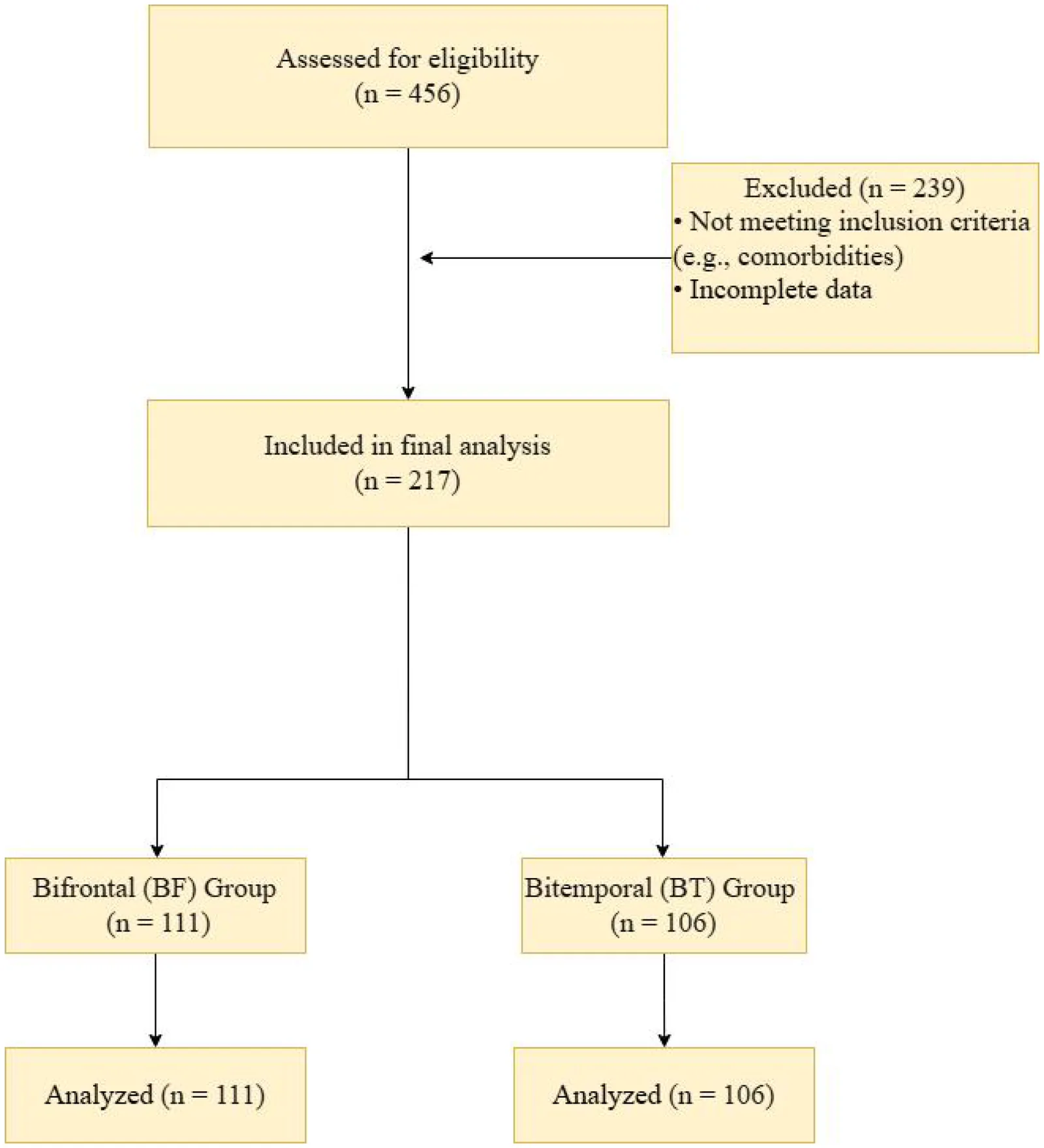
Flowchart of patient screening and enrollment.

We excluded patients with a diagnosis of schizophrenia spectrum disorders, bipolar disorder, or organic brain diseases to ensure a homogenous sample of unipolar depression. Additionally, patients who received mixed electrode placements during the same treatment course (e.g., switching from BF to BT due to non-response) were excluded to strictly isolate the effects of each modality. Specifically, a total of 14 patients were excluded for this reason; all 14 patients were switched from BF to BT due to inadequate clinical response (defined as <25% reduction in HAMD-24 after 6 sessions). Patients with incomplete clinical data regarding the primary outcome measures were also excluded from the final analysis. The study flow diagram is shown in **Figure 1**.

### 2.2 ECT procedures

ECT was administered in a dedicated post-anesthesia care unit equipped with standard monitoring capabilities. Patients were pre-oxygenated with oxygen. Anesthesia was induced with intravenous propofol (1.5–2.0 mg/kg), and muscle relaxation was achieved with succinylcholine (0.5–1.0 mg/kg). Ventilation was assisted with a bag-valve-mask during the procedure. Treatments were administered twice weekly, consistent with standard ECT practice as outlined in the NICE guidance (19). A Thymatron System IV device (Somatics, LLC, Lake Bluff, IL, USA) was used for all treatments, utilizing brief-pulse stimulation (pulse width 0.5–1.0 ms). Patients were categorized into two groups based on the specific electrode placement technique documented in the procedure notes. In the Bitemporal (BT) Group, electrodes were applied bifrontotemporally, with the center of each electrode placed approximately 2.5 cm above the midpoint of an imaginary line connecting the tragus of the ear and the lateral canthus of the eye. In the Bifrontal (BF) Group, electrodes were placed on the forehead, with the center of each electrode positioned 5 cm apart and approximately 2.5 cm above the outer canthus of each eye. Electrode placement was not randomized but determined by the treating psychiatrist in consultation with guardians. No formal institutional guideline or algorithm governed this choice. In practice, clinicians tended to favor bitemporal placement for patients with psychotic features, high imminent suicide risk, or prior treatment non-response—situations where rapid, robust effect was prioritized—and bifrontal placement when cognitive preservation was a primary concern. This non-randomized allocation introduces potential selection bias, as patients with greater clinical severity may have been disproportionately assigned to the BT group (22).

The initial stimulus dose was determined using the “half-age” method or empirical titration. During subsequent sessions, stimulus intensity was adjusted according to seizure adequacy, including motor seizure duration, EEG seizure characteristics, and postictal suppression. In cases of inadequate seizures, the stimulus dose was increased in the following session according to the routine institutional ECT protocol. All dose adjustments were performed by experienced ECT clinicians using consistent clinical criteria throughout the study period. A therapeutic seizure was defined as a motor seizure duration of at least 25 seconds or an EEG seizure duration of at least 30 seconds with robust postictal suppression (23). Identical dosing and titration protocols were applied to both electrode placement groups to minimize confounding by stimulus intensity. Baseline psychotropic utilization is summarized in **Table 1**. While we encouraged tapering or discontinuation of benzodiazepines prior to ECT to minimize interference, their use was maintained in a subset of patients for clinical management of severe agitation and was systematically included in the multivariate analysis. Daily dosages of antidepressants and antipsychotics were converted into fluoxetine equivalents and risperidone equivalents, respectively.

**Table 1:** Baseline demographic and clinical characteristics.

Characteristic	Bifrontal (BF) (n=111)	Bitemporal (BT) (n=106)	Statistic	P-value
Age (years)	15.6 ± 1.5	15.4 ± 1.6	t = 0.95	0.342
Sex (Female), n (%)	77 (69.4%)	76 (71.7%)	χ² = 0.14	0.708
Duration of illness (months)	14.3 ± 9.0	15.6 ± 10.0	t = -1.03	0.306
Number of depressive episodes	1.4 ± 0.6	1.5 ± 0.7	t = -0.91	0.366
Prior ECT treatment (Yes), n (%)	12 (10.8%)	13 (12.3%)	χ²=0.08	0.777
Psychotic features at baseline (Yes), n (%)	18 (16.2%)	22 (20.8%)	χ²=0.58	0.445
No. of Sessions	9.8 ± 1.8	10.1 ± 2.1	t = -1.12	0.264
Baseline HAMD-24	32.7 ± 3.8	31.8 ± 3.3	t = 1.77	0.078
Baseline BSSI	22.8 ± 6.5	23.5 ± 5.8	t = -0.83	0.407
Benzodiazepines	42 (37.8%)	45 (42.5%)	χ² = 0.49	0.484
Antipsychotic dose(Risperidone-eq, mg/day)	2.8 ± 1.5	3.1 ± 1.8	t = -1.33	0.185
Antidepressant dose (Fluoxetine-eq, mg/day)	38.5 ± 12.4	40.2 ± 14.1	t = -0.94	0.348
Antidepressant class, n (%)			χ²=0.79	0.673
SSRI	80 (72.1%)	76 (71.7%)		
SNRI	19 (17.1%)	17 (16.0%)		
other	12 (10.8%)	13 (12.3%)		

Continuous variables are presented as mean ± standard deviation and were compared using independent samples t-test; categorical variables are presented as n (%) and were compared using chi-square test.

Right unilateral (RUL) electrode placement was not included as a comparator in this study. During the study period, RUL ECT was rarely used at our center. As only a small number of patients received RUL ECT, the sample was insufficient to support a meaningful comparison. Therefore, the present analysis is confined to the BF versus BT comparison.

### 2.3 Outcome measures

The primary efficacy outcome was the reduction in depressive symptoms assessed by the 24-item Hamilton Depression Rating Scale (HAMD-24) (27). Assessments were conducted at baseline (before the first ECT session) and at the end of the ECT course. “Response” was defined as a reduction of at least 50% in the baseline HAMD-24 score. “Remission” was defined as a final HAMD-24 score of 8 or lower, indicating a return to euthymia. Given the high prevalence of suicide risk in this population, we also utilized the Beck Scale for Suicide Ideation (BSSI) as a secondary efficacy measure to specifically evaluate the anti-suicidal effects of the treatment (28). Safety outcomes focused on subjective cognitive function and immediate physical recovery. Subjective memory complaints were assessed daily by treating psychiatrists via standardized verbal inquiries (e.g., “Have you experienced difficulty remembering recent events?” or “Do you feel confused about what happened during your hospital stay?”). A single affirmative report during the entire ECT course—whether spontaneously reported by the patient or elicited by physician inquiry—was recorded as “Yes”. The treating psychiatrists who conducted these assessments were not blinded to electrode placement, as blinding was not feasible in this retrospective study design. Consequently, this non-blinded, subjective assessment is prone to expectancy bias and does not substitute for standardized neuropsychological testing. Post-ictal recovery time was objectively measured as the time interval in minutes from the cessation of the seizure to the return of purposeful movement and full orientation to time, place, and person. Other common adverse events, including post-ictal headache and nausea, were extracted from routine nursing and medical records based on spontaneous patient reports.

### 2.4 Statistical analysis

Statistical analyses were performed using SPSS version 26.0 (IBM Corp., Armonk, NY, USA). Continuous variables, such as age, illness duration, scores on HAMD-24 and BSSI, and recovery time, were presented as mean ± standard deviation (SD). Normality was assessed using the Shapiro–Wilk test. Comparisons between the BF and BT groups were conducted using the independent samples t-test for normally distributed variables; otherwise, the Mann–Whitney U test was used. Categorical variables, including sex, response rates, remission rates, and the incidence of adverse events, were presented as counts and percentages and were compared using the Chi-square test or Fisher’s exact test where appropriate. Effect sizes were reported as odds ratios (OR) with 95% confidence intervals for categorical outcomes and Cohen’s d for continuous outcomes. A two-tailed *p*-value of less than 0.05 was considered statistically significant. As this was a retrospective study utilizing all available eligible cases within the specified timeframe, a formal *a priori* sample size calculation was not performed; furthermore, no adjustments for multiple comparisons were conducted, which is acknowledged as a study limitation. All primary efficacy and safety outcome measures (HAMD-24, BSSI, memory complaints, recovery time) were documented as standard clinical practice, resulting in no missing data for these variables. For the rare instance of missing data in secondary variables (e.g., headache, nausea), complete-case analysis was applied. Furthermore, a multivariate logistic regression model was constructed to identify independent predictors of subjective memory complaints. The model included electrode placement, age, baseline HAMD-24 score, number of ECT sessions, concurrent benzodiazepine use, and equivalent doses of antidepressants and antipsychotics (fluoxetine and risperidone equivalents, respectively) as independent variables.

## 3 Results

### 3.1 Demographic and clinical characteristics

A total of 456 adolescents were screened for eligibility. After applying the exclusion criteria, a final cohort of 217 adolescents was included in the analysis. The cohort was divided into the Bifrontal (BF) group (n = 111) and the Bitemporal (BT) group (n = 106). The baseline demographic and clinical characteristics are summarized in **Table 1**. The two groups were well-matched with no statistically significant differences in age (p = 0.342), sex distribution (p = 0.708), duration of illness (p = 0.306), number of depressive episodes(p =0.366), prior ECT history(p = 0.777), psychotic features(p = 0.445), and antidepressant class(p = 0.673). The mean baseline HAMD-24 score was 32.7 ± 3.8 in the BF group and 31.8 ± 3.3 in the BT group (p = 0.078), indicating a population with severe depression. Similarly, the baseline suicide risk, as measured by the BSSI, was comparable between the two groups (22.8 ± 6.5 vs. 23.5 ± 5.8, p = 0.407). The total number of ECT sessions received during the course was also similar (9.8 ± 1.8 vs. 10.1 ± 2.1, p = 0.264).

### 3.2 Clinical efficacy outcomes

The clinical efficacy outcomes are summarized in **Table 2**. Both electrode placements were followed by rapid and substantial improvement in depressive symptoms. At the end of the treatment course, the mean HAMD-24 scores dropped in both groups to 11.6 ± 5.6 (BF) and 11.5 ± 4.4 (BT), with no significant difference between the groups (p = 0.887; Cohen’s d = 0.02). Regarding the categorical outcomes, the BF group achieved a response rate of 85.6% (95/111) compared to 82.1% (87/106) in the BT group, but it did not reach statistical significance (χ² = 0.50, p = 0.478). Remission rates were also comparable, with 33.3% of the BF group and 33.0% of the BT group achieving full remission (χ² < 0.01, p = 0.963; OR = 0.93, 95% CI 0.52–1.66). Furthermore, both modalities proved highly effective in resolving suicidal ideation. The BSSI scores decreased significantly from baseline in both groups. There was no statistically significant difference in the end-treatment BSSI scores between the BF group (6.5 ± 2.8) and the BT group (7.1 ± 3.5) (p = 0.215; Cohen’s d = 0.19), suggesting that both placements are equally capable of managing acute suicidality (29).

**Table 2:** Comparison of clinical efficacy outcomes.

Outcome measures	Bifrontal (BF) (n=111)	Bitemporal (BT) (n=106)	Statistic	P-value
End HAMD-24	11.6 ± 5.6	11.5 ± 4.4	t = 0.14	0.887
Reduction rate (%)	64.6 ± 16.1	64.6 ± 15.9	t = 0.01	0.992
Response rate, n (%)	95 (85.6%)	87 (82.1%)	χ² = 0.50	0.478
Remission rate, n (%)	37 (33.3%)	35 (33.0%)	χ² < 0.01	0.963
End BSSI score	6.5 ± 2.8	7.1 ± 3.5	t = -1.24	0.215

Response was defined as ≥50% reduction in HAMD-24 score; remission was defined as HAMD-24 score ≤8. Continuous variables were compared using independent samples t-test; categorical variables were compared using chi-square test.

### 3.3 Safety and adverse events

While efficacy outcomes were similar, the safety profiles of the two modalities diverged significantly (**Table 3**). We observed a potentially lower incidence of subjective memory complaints in the BF group. Only 28.8% (32/111) of adolescents in the BF group reported subjective memory impairment, in stark contrast to 67.9% (72/106) in the BT group. This difference was highly statistically significant (χ² = 33.17, p < 0.001; OR = 0.20, 95% CI 0.11–0.36).

**Table 3:** Comparison of safety profile.

Adverse events	Bifrontal (BF) (n=111)	Bitemporal (BT) (n=106)	Statistic	P-value
Subjective memory complaints	32 (28.8%)	72 (67.9%)	χ² = 33.17	< 0.001
Recovery time (min)	23.7 ± 7.7	33.4 ± 9.9	t = -8.07	< 0.001
Headache	51 (45.9%)	55 (51.9%)	χ² = 0.78	0.378
Nausea	14 (12.6%)	16 (15.1%)	χ² = 0.28	0.597

Recovery time is presented as mean ± standard deviation and was compared using independent samples t-test; incidence of adverse events is presented as n (%) and was compared using chi-square test.

Objective measures of recovery also favored the BF group. Patients treated with BF ECT regained orientation and purposeful movement significantly faster than those treated with BT ECT (mean recovery time: 23.7 ± 7.7 minutes vs. 33.4 ± 9.9 minutes; t = -8.07, p < 0.001; Cohen’s d = 1.12). There were no significant differences in the incidence of other somatic side effects, such as post-ictal headache (45.9% vs. 51.9%, p = 0.378) or nausea (12.6% vs. 15.1%, p = 0.597), suggesting that the safety advantage of BF placement is specific to cognitive and neurological recovery.

### 3.4 Multivariate logistic regression for subjective memory complaints

To further investigate whether the cognitive advantage of BF placement was independent of potential confounders, a multivariate logistic regression was performed (**Table 4**). After adjusting for age, baseline depression severity (HAMD-24), total number of ECT sessions, and concurrent psychotropic medications (including benzodiazepine use and equivalent dosages of antidepressants and antipsychotics), electrode placement remained a significant independent predictor of subjective memory complaints. Specifically, patients receiving BF-ECT were significantly less likely to report memory impairment compared to those receiving BT-ECT (Adjusted OR = 0.21, 95% CI: 0.11–0.39, p < 0.001). Additionally, the concurrent use of benzodiazepines was identified as an independent risk factor for memory complaints (Adjusted OR = 2.65, 95% CI: 1.32–5.31, p = 0.006). Although tapering of benzodiazepines was encouraged prior to ECT, ongoing use was necessary in some patients for the management of severe agitation, supporting its inclusion as a covariate in the regression model.

**Table 4:** Multivariate logistic regression analysis for subjective memory complaints.

Predictors	Adjusted OR	95% CI	P-value
Electrode placement (BF vs. BT) *(Ref: BT)*	0.21	0.11 – 0.39	< 0.001
Age (years)	0.97	0.83 – 1.14	0.785
Baseline HAMD-24 score	1.04	0.95 – 1.15	0.312
Number of ECT sessions	1.10	0.95 – 1.28	0.188
Benzodiazepines (Yes vs. No)	2.65	1.32 – 5.31	0.006
Antidepressant dose (Fluoxetine-eq, mg/day)	1.01	0.98 – 1.04	0.510
Antipsychotic dose (Risperidone-eq, mg/day)	1.05	0.88 – 1.25	0.425

aOR, adjusted odds ratio; CI, confidence interval; HAMD-24, 24-item Hamilton Depression Rating Scale. The multivariate model adjusted for age, baseline HAMD-24 scores, total number of ECT sessions, concurrent benzodiazepine use (binary), and equivalent dosages of antidepressants (Fluoxetine-eq) and antipsychotics (Risperidone-eq).

## 4 Discussion

This study, through a retrospective cohort analysis, suggests that bifrontal ECT may be as effective as traditional bitemporal ECT regarding antidepressant and anti-suicidal outcomes. In addition, BF ECT was associated with fewer subjective memory complaints and shorter post-ictal recovery time. However, given the retrospective, non-randomized design and the non-blinded, subjective nature of the cognitive assessment, these safety findings should be regarded as exploratory and hypothesis-generating rather than confirmatory evidence of cognitive superiority. We found no statistical differences between the two groups in the absolute reduction of HAMD-24 scores, percentage reduction, or remission rates, with the BF group even showing a numerically higher response rate. Conversely, the incidence of subjective memory complaints was more than halved (OR = 0.20) in the BF group, and the mean postictal recovery time was shortened by nearly 10 minutes (Cohen’s d = 1.12).

In line with findings from systematic reviews and population-based studies, both BF and BT placements were associated with rapid antidepressant and anti-suicidal effects in our sample, with response rates generally consistent with previously reported ranges of 70%-82% for adolescent depression treated with bilateral ECT (24). However, remission rates were approximately 33%, somewhat lower than rates reported in some adult and adolescent ECT studies. Several factors may account for this. The cohort had high baseline BSSI scores (above 22 in both groups), suggesting a high overall suicide risk. Blanken et al. (2025) reported that baseline suicidal ideation may reduce the likelihood of ECT remission (OR = 0.75) (25), and Rönnqvist et al. (2022) observed a remission rate of 27.2% in patients with suicidal ideation, a range consistent with our findings (26). Furthermore, the average of approximately 10 sessions may have been insufficient for some patients to reach full remission. Additionally, ECT discontinuation was guided by clinical judgment rather than a standardized protocol; some patients may have stopped treatment once meaningful improvement was achieved.

Our findings extend evidence from the adult literature, where randomized controlled trials have demonstrated equivalent antidepressant efficacy and superior cognitive outcomes for BF compared with BT ECT (15, 16). While existing pediatric ECT data are largely limited to small case series (13), our study provides comparative evidence specifically for the adolescent population at a critical neurodevelopmental stage.

Although the clinical importance of subjective memory complaints in this population is well-documented, and our findings in this domain remain clinically informative (13, 14), our cognitive safety findings should not be equated with results from formal neuropsychological testing. The non-standardized, non-blinded nature of the assessment introduces inherent information bias that cannot be fully corrected by the statistical adjustments we performed. Subjective memory discomfort is the most prominent and patient-important adverse effect in adolescent ECT, even when objective cognitive deficits are absent or minimal (14), and reducing such complaints may improve treatment adherence in this population (30).

These limitations notwithstanding, the observed differences between BF and BT placements warrant consideration of their relative clinical utility. However, it is important to note that the present study did not include a RUL ECT group. Consequently, we are unable to draw any conclusions regarding the comparative efficacy or cognitive safety of RUL versus bilateral placements (BF or BT) in this specific adolescent population. Our discussion is therefore strictly focused on the direct comparison between BF and BT electrode placements. Importantly, the comparable remission rates combined with the substantially lower rate of subjective memory complaints in the BF group suggest that BF ECT offers a more favorable risk–benefit profile for adolescent MDD. The observed antidepressant and anti-suicidal outcomes did not differ significantly between BF and BT ECT, while the cognitive burden—a critical concern in this developmental population—was significantly lower. However, this interpretation should be viewed in light of the potential selection bias inherent in the non-randomized design: patients with greater clinical severity were more likely to receive BT ECT. If BT-treated patients were indeed more severely ill, the comparable remission rates could alternatively suggest greater efficacy of BT placement. Future randomized trials are needed to definitively resolve this question.

The adolescent brain exhibits higher plasticity and differential sensitivity to stimulation. Our results indicate that the prefrontal cortex remains a key target for ECT efficacy, while bypassing the temporal lobe pathway may translate into substantial cognitive benefits. This may challenge a common clinical notion that the more potent bitemporal placement should be the default for severe, high-risk adolescent patients. Our data showed no significant between-group difference in the reduction of suicidal ideation, including among patients with severe clinical presentations, suggesting that the continued preferential use of the cognitively riskier bitemporal method may warrant reconsideration in clinical practice (31). The lower rate of subjective memory complaints observed with BF ECT could be viewed as consistent with the hippocampal-sparing hypothesis. Computational modeling studies have suggested that bifrontal placement directs current primarily to prefrontal and orbitofrontal regions, with reduced exposure of temporal lobe structures compared to bitemporal placement (10). However, the present study did not include neuroimaging or objective neurocognitive measures, and these mechanistic considerations remain speculative; they are offered as possible explanations rather than conclusions directly supported by our data. For adolescent patients, memory and learning functions are central to academic achievement. Treatment-induced memory difficulties can lead to “secondary academic anxiety” and even act as psychosocial triggers for depressive relapse. Whether the cognitive preservation associated with BF-ECT may translate into better long-term functional outcomes remains to be determined in prospective longitudinal studies. Similarly, the shorter post-ictal recovery time in the BF group could be hypothesized to reflect milder or more transient post-ictal network inhibition, although this interpretation remains tentative in the absence of electrophysiological or neuroimaging data (32).

Based on these findings, we propose that bifrontal electrode placement may be considered a reasonable initial choice for adolescents with MDD requiring ECT, particularly when cognitive preservation is a clinical priority. Given the retrospective non-randomized design of this study, BT ECT may still be considered in clinical scenarios where BF ECT yields insufficient response. This strategy may help optimize the risk-benefit profile of adolescent ECT, preserving cognitive function in the developing brain while maintaining rapid antidepressant and anti-suicidal efficacy (33).

Several limitations of this study must be acknowledged. First, the non-randomized allocation of electrode placement introduces potential confounding by baseline severity: patients with greater acuity or suicide risk were more likely to receive BT ECT. This may bias both efficacy and safety estimates despite statistical adjustment. Detailed ECT parameters such as final stimulus charge, EEG seizure duration, and post-ictal suppression index were also not consistently recorded; and the lack of blinding may have introduced expectation bias in subjective cognitive reports. In addition, although benzodiazepine use was comparable between groups, detailed data on dose and timing relative to ECT sessions were not available, which may have introduced residual confounding. Second, cognitive safety was evaluated using subjective memory complaints rather than standardized neuropsychological testing. Third, we did not adjust for multiple comparisons, although key safety findings remained highly significant. Fourth, this study focused on acute outcomes during the ECT course. Data on longer-term outcomes such as relapse rates, durability of response, cognitive recovery, school and social functioning, and longer-term safety were beyond the scope of this retrospective design. This is a relevant limitation given the developmental importance of these domains in adolescents. Fifth, although known bipolar disorder was excluded, diagnostic uncertainty is common in adolescent MDD, as some patients may later convert to bipolar disorder. Such misclassification could potentially influence ECT response patterns or adverse event profiles, and this should be considered when interpreting the findings. Sixth, excluding patients who switched from BF to BT due to non-response (n=14) may have introduced survivor bias. Seventh, the high baseline suicidal ideation of the cohort, non-standardized ECT termination criteria, and limited treatment intensity may have contributed to the relatively low remission rates. Finally, the absence of a right unilateral ECT comparison group limits conclusions to the BF versus BT comparison.

## 5 Conclusion

In adolescents with MDD, bifrontal ECT showed short-term antidepressant and anti-suicidal outcomes comparable to those of bitemporal ECT in this retrospective cohort. BF ECT appeared to be associated with fewer subjective memory complaints (as assessed by spontaneous patient reports and daily physician inquiries) and shorter post-ictal recovery time. These two endpoints, while clinically relevant in routine practice, do not capture the full spectrum of cognitive function; standardized neuropsychological assessment would be required for a comprehensive comparison. Given the retrospective design and non-random treatment allocation, these findings should be interpreted cautiously and confirmed in prospective controlled studies.

## Data Availability

The original contributions presented in the study are included in the article/supplementary material. Further inquiries can be directed to the corresponding author.
